# New Ideas in Obstetrics from America: Current Views on Cæsarean Section

**Published:** 1917-02-03

**Authors:** 


### NEW IDEAS IN OBSTETRICS FROM AMERICA.

### Current Views on Cæsarean Section.

In the state of preoccupation with war medicine and surgery in which the medical profession of Britain exists, little research or advance other than of a military character is either possible or desirable. In the United States, however, there is a very lively—even adventurous—spirit of progress abroad; and a study of recent American literature shows that, amongst other subjects, an enormous amount of attention has been bestowed upon Cæsarean section lately over there. The ever-present tendency is towards a continual expansion of the indications for that operation, already far more widely practised in the New World than in the Old.

### Cæsarean Section for Breech Presentations.

As an instance of the lengths to which the "forward" school of American obstetricians will go, it is interesting to note that J. T. Williams, of Boston, advocates Cæsarean section for most cases of breech presentation. This novel proposal is criticised somewhat severely by a New York authority, Ross McPherson, and does not seem to have commanded any great degree of assent as yet; but clearly the mere suggestion of such a line of treatment is evidence of a more expansive attitude towards operative delivery through the abdomen than has hitherto been adopted here. McPherson has analysed a mass of statistics from a New York maternity institution, with a view to reaching a sound basis of comparison between the results of breech delivery *per vias naturales* and *per abdomen*. He finds as a result that, when disturbing factors such as placenta prævia, eclampsia, and other conditions have been eliminated, the maternal death-rate for breech presentations is no higher than for those of the vertex. For the children the mortality is, of course, much higher; breech presentations are variously estimated to result in 10 to 30 per cent. of fœtal deaths. McPherson puts it down at 9.5 per cent. in pregnancies that have gone to full term; curiously enough, he found little difference in this respect between primiparæ and multiparæ, whereas most statisticians hold that among the former the (fœtal) death-rate is much the higher. He states that even in the best and most experienced hands there is still a maternal mortality of from 2 to 4 per cent. from Cæsarean section, an estimate which is doubtless true of institutional practice wherein many of the patients are already contaminated with sepsis on admission, but is much too high for clean elective cases when eclampsia and similar dangerous conditions are excluded. He does not say that Cæsarean section is never advisable for breech cases, but that it is "a too radical and rarely necessary operation" for routine adoption.

### Rupture of the Cæsarean Scar.

Drs. Bell, of Detroit, and Rongy, of New York, have independently read careful papers on Rupture of the Cæsarean Scar in subequent pregnancies. Both of them have had experience of this complication, and both of them have delved into the literature concerning it. Dr. Bell holds that if the puerperium following the first section has not been afebrile, gestation should never be allowed to go to term on a subsequent occasion; labour should be induced at least a fortnight beforehand. Dr. Rongy says that spontaneous rupture of a Cæsarean scar occurs in about 3 per cent. of cases, usually during labour, but also occasionally during the last six weeks of pregnancy. He knows of no way of foretelling this accident, and attaches little importance to afebrile convalescence from the primary operation; not even the most refined suturing will guarantee immunity from this complication. The mortality rate of repeated Cæsarean section is less than that of the primary operation, for which reason among others he says that no patient who has once had section should be allowed to go through a tedious or long labour, for fear of rupture of the old scar. As a general rule, he finds that about 75 per cent. of those who have had Cæsarean section are delivered in the same way during subsequent labours. Finally, he says emphatically that Cæsarean section is being performed unnecessarily and too frequently in America, and by operators who lack the necessary surgical experience and technique. In this country the same criticism, in our judgment, most certainly does not hold good; if anything, our obstetricians are over-conservative and have not been quite so quick to appreciate the great value of immediate Cæsarean section for one or two conditions, such as central placenta prævia and severe eclampsia. It is largely because Cæsarean section in Britain has been used nearly exclusively for contracted pelvis, fibroids, and similar conditions of a more or less permanent character that our literature contains fewer cases of rupture of the scar; because in most of such cases the condition which called for the first section calls equally for a second one, whereas placenta prævia and eclampsia are far less likely to recur in subsequent pregnancies.

### Gynæcological Pharmacology.

A valuable piece of research in the pharmacology of various drugs reputed to have valuable properties in uterine disorders has been published by Drs. Pilcher, Delzell, and Burman. Their experiments were conducted on the guinea-pig, so that some caution is necessary in applying them to human medicine. Many of the drugs mentioned in this report are scarcely used in Britain, and are indeed unknown even by name save to a handful of homœopathists. These investigators find that both the infusion and the fluid extract of the following are quite inert: black haw (root and stem), cramp bark, squaw vine, chestnut bark, false unicorn, passion flower, blessed thistle, St. Mary's thistle, motherwort—only the first, sixth, and last of these have any appreciable vogue in this country.

Four drugs have some power of inhibiting uterine contractions; unicorn root, pulsatilla, Jamica dogwood, and figwort. Valerian and lady's slipper have a much less pronounced similar action; and wild yam, life root, and skull-cap are almost inert. The only drug which excites uterine action in the guinea-pig is blue cohosh, which puts the uterus practically in a state of tetanus.

